# Applied anatomical study on suprascapular nerve protection in reverse total shoulder arthroplasty

**DOI:** 10.1186/s13018-020-02061-2

**Published:** 2020-11-11

**Authors:** Jianfeng Li, Junlin Zhou, Dong Wang, Dacun Li, Wentong Zhang

**Affiliations:** 1 Department of Orthopaedics, Beijing Chaoyang Hospital of Capital Medical University, No. 8 of Gongren Tiyuchang Nanlu, Chaoyang District, Beijing, 100020 PR China; 2 Department of Upper Limb Surgery, Beijing Shunyi District Hospital, No. 3 of Guangming Nanjie, Shunyi District, Beijing, 101300 PR China

**Keywords:** Reverse total shoulder arthroplasty, Glenoid cavity, Morphology, Three-dimensional reconstruction, Suprascapular nerve

## Abstract

**Background:**

This study aimed to investigate the three-dimensional (3D) anatomical relationship between the suprascapular nerve and scapula, and the method of protecting the suprascapular nerve in reverse total shoulder arthroplasty (RTSA)

**Methods:**

In the present study, 12 fresh adult cadaver shoulder specimens were dissected. X-ray and computed tomography (CT) were used to investigate the 3D scapular and suprascapular nerve images.

**Results:**

The results revealed that the best fitting baseplate diameter was 24.73 ± 1.56 mm. Furthermore, the baseplate diameter correlated with the glenoid cavity width. After the osteotomy, a simulated screw placement on the baseplate was performed. The dangerous area for the posterior screw placement was at the angle between the upper edge and transverse axis exceeding 38° and between the lower edge and transverse axis exceeding 76°. The distance between the nearest point of the nerve and osteotomy plane was 15.38 ± 2.02 mm, and the angle between the projection point of the nearest point and transverse axis was 27.33 ± 7.96°, which was the dangerous area for retractor placement. The suitable angle between the superior screw and longitudinal axis was 21.67 ± 13.27°, and the suitable superior screw length was 34.66 ± 2.41 mm.

**Conclusion:**

In RTSA, the baseplate size correlates with the glenoid cavity width. The relationship between the screw and suprascapular nerve and retractor placement position should be carefully considered to avoid damaging the suprascapular nerve.

## Background

Reverse total shoulder arthroplasty is an essential method to treat various severe shoulder joint diseases [1–3]. Furthermore, reverse total shoulder arthroplasty is good for reconstructing the shoulder joint, improving shoulder joint function, and relieving pain [4]. Recently, reverse total shoulder arthroplasty has had wider indications than anatomical shoulder arthroplasty in clinics [5].

Due to the non-anatomical design of reverse shoulder prostheses, the incidence of complications related to peripheral nerve injury in reverse shoulder arthroplasty remains significantly higher than that in anatomical shoulder arthroplasty. The incidence of peripheral nerve complications of reverse shoulder arthroplasty ranges within 0.6–3.6% [6], while that of subclinical peripheral nerve complications can reach as high as 47.8% [7], which were mainly indicated by axillary nerve and suprascapular nerve injuries.

The suprascapular nerve is closely bound to the bone surface in the supraspinous fossa, in the infraspinous fossa, and around the spinoglenoid notch [8]. However, there is a risk of suprascapular nerve injury in reverse shoulder arthroplasty in the fixation of glenoid cavity prosthesis components [9, 10]. The suprascapular nerve is affected by excessively long screws penetrating the contralateral cortex [11, 12]. The suprascapular nerve is located in the deep muscle layer above and behind the glenoid cavity, which cannot be exposed during surgery. Therefore, the evaluation of the dangerous area of the suprascapular nerve in the glenoid cavity and identifying a safe screw length are necessary to reduce suprascapular nerve injuries.

Although anatomical studies on the glenoid cavities and suprascapular nerves of cadaveric specimens have been conducted to determine the safe area to avoid suprascapular nerve injury during reverse shoulder arthroplasty [12], these studies were limited to the surface measurement of the glenoid cavity and its anatomical relationship with the suprascapular nerve. Due to the soft-tissue covering, the three-dimensional (3D) images of the suprascapular nerve and glenoid cavity could not be formed, the simulated osteotomy could not be performed, and the glenoid cavity sections could not be measured. Therefore, the aim of our study was to investigate the three-dimensional (3D) anatomical relationship between the suprascapular nerve and scapula, which could provide a reference for protecting the suprascapular nerve in reverse total shoulder arthroplasty (RTSA).

## Methods

### Subjects

In the present study, 12 fresh adult cadaver shoulder specimens were dissected. The average age of these specimens was 79 years old (range, 68–89 years old). The specimens included the intact scapula, upper half of the humerus, clavicle, intact shoulder joint, muscles of the shoulder, muscles of the arm, and chest wall muscle. The cadaveric specimens were intact and had no shoulder joint deformity, defect, shoulder surgery history, or trauma history. The present study was conducted in accordance with the Declaration of Helsinki and approved by the ethics committee of our hospital (QR-112-045 No.113).

### Methods

**Fig. 1:**
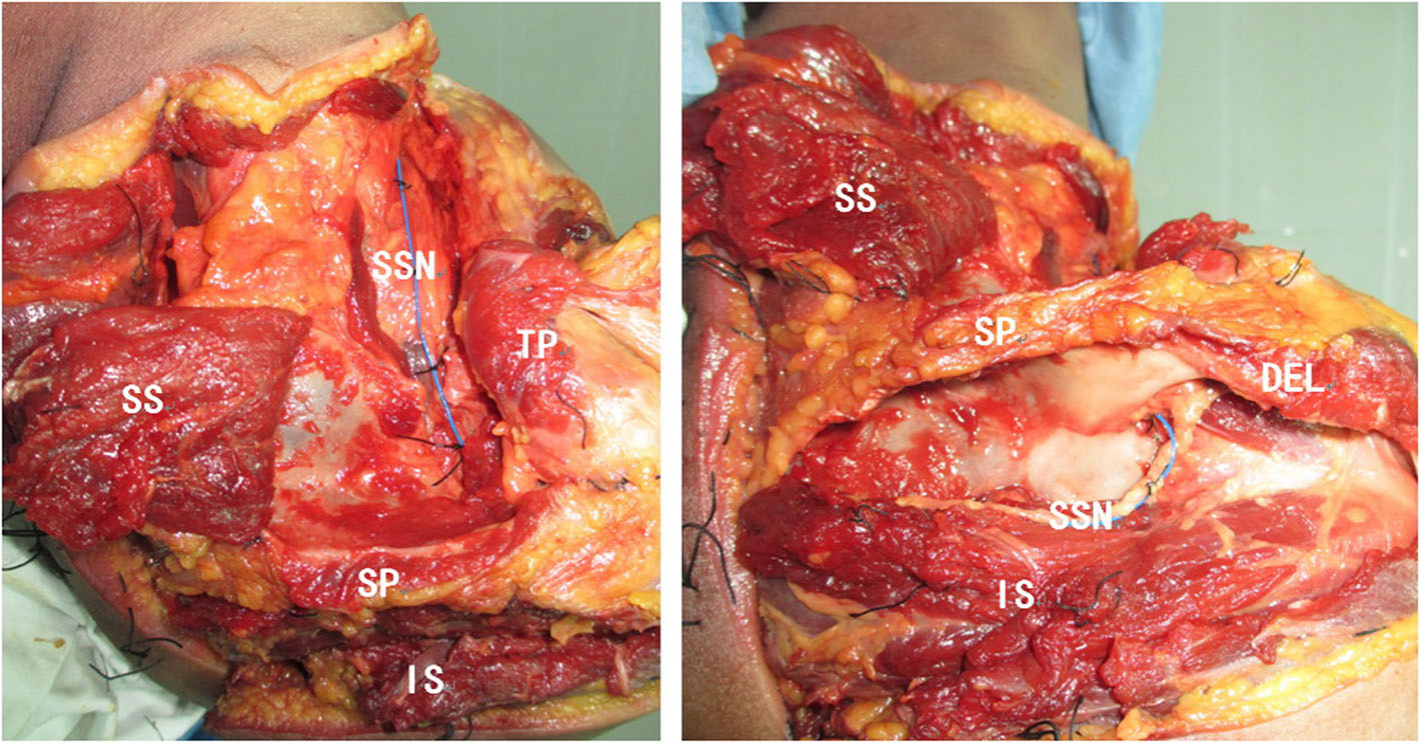
Anatomy of the suprascapular nerve. SS, supraspinatus; IS, infraspinatus; SP, scapular spine; SSN, suprascapular nerve; TP, triceps; DEL, deltoid

The suprascapular nerve was exposed using the modified Judet approach [13]. After the subcutaneous tissue was separated, the trapezius was cut off at the end point of the acromion and scapular spine and turned inward to expose the supraspinatus and infraspinatus. Then, the attachment points of these two muscles at the medial edge were stripped off, and these were turned over to expose the suprascapular transverse ligament, scapular notch, and spinoglenoid notch. Careful dissection was performed under a × 4 magnifier to expose the suprascapular nerve and its branches. A developing thread (an X-ray-detectable medical barium thread) of 0.8 mm in diameter was sutured to the suprascapular nerve for marking, with one stitch for each 1 cm (Fig. 1). Attention was given to maintaining the original position of the nerve during the suture. Finally, the muscle layers were repaired, and the incision was closed.

The shoulder joint specimens were scanned using a Siemens CT scanner (120 kVp; 320 mA; 512 × 512 matrix; slice thickness, 0.6 mm). The scan data were saved in DICOM format and imported into the Mimics 17.0 software (Materialize, Leuven, Belgium) for threshold segmentation and region growing.

In the present study, the natural course of the suprascapular nerve was marked with X-ray-detectable (radiopaque) medical barium threads, the specimens were scanned using computed tomography (CT), and the Mimics software was used to perform the 3D reconstruction, in order to determine the 3D anatomical relationship between the suprascapular nerve and scapula. The osteotomy and baseplate placement were simulated to clarify the relationship between the glenoid cavity shape and baseplate size and define the safe area for the glenoid cavity and safe distance for screws, in order to provide a reference for suprascapular nerve protection in clinical reverse shoulder arthroplasty.

### The measurement process

#### Step a

**Fig. 2:**
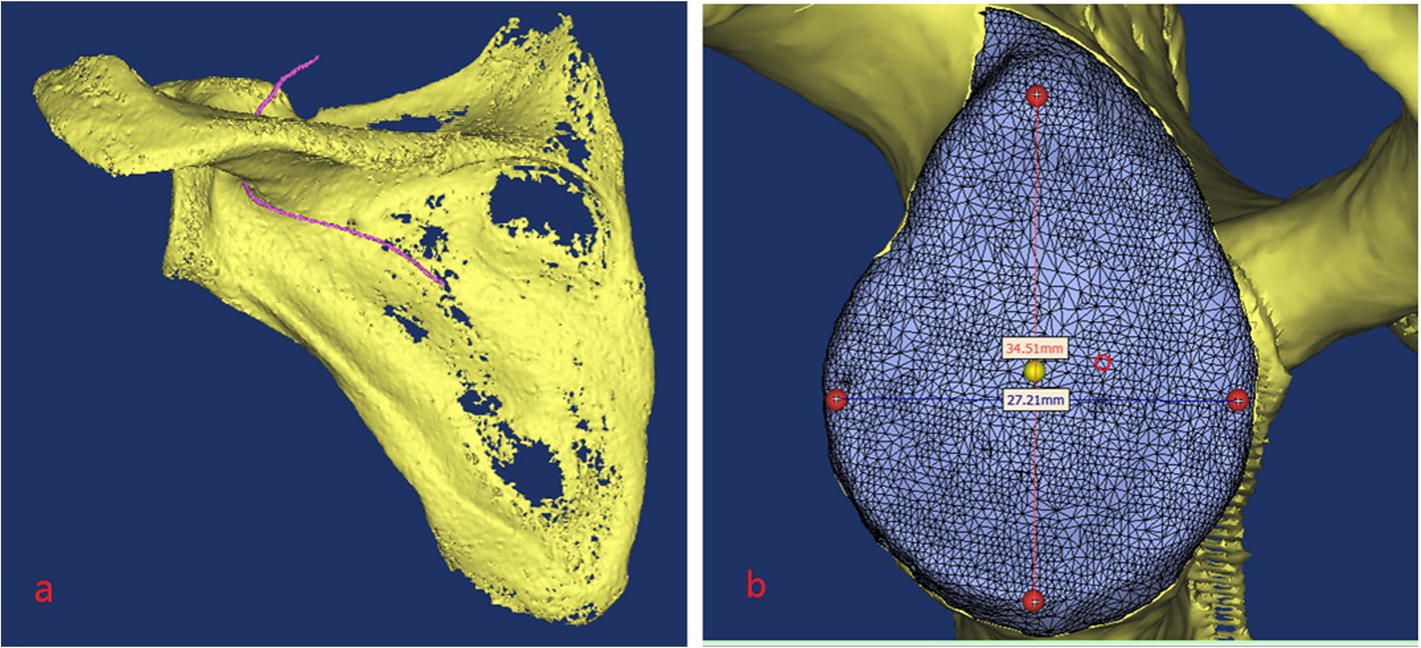
3D reconstruction and glenoid cavity measurement

The 3D reconstruction was performed using the scan data, and the 3D images of the scapula and suprascapular nerve were formed (Fig. 2a).

#### Step b

The glenoid cavity was measured. The height (*h*) and width (*w*) of each glenoid cavity were measured by setting the upper, lower, anterior, and posterior poles. The glenoid cavity surface center was determined: simulated segmentation was performed, the glenoid cavity fossa was gridded, and the center of gravity of the glenoid cavity fossa was calculated as point O of the center of the glenoid cavity surface [14] (Fig. 2b).

#### Step c

**Fig. 3:**
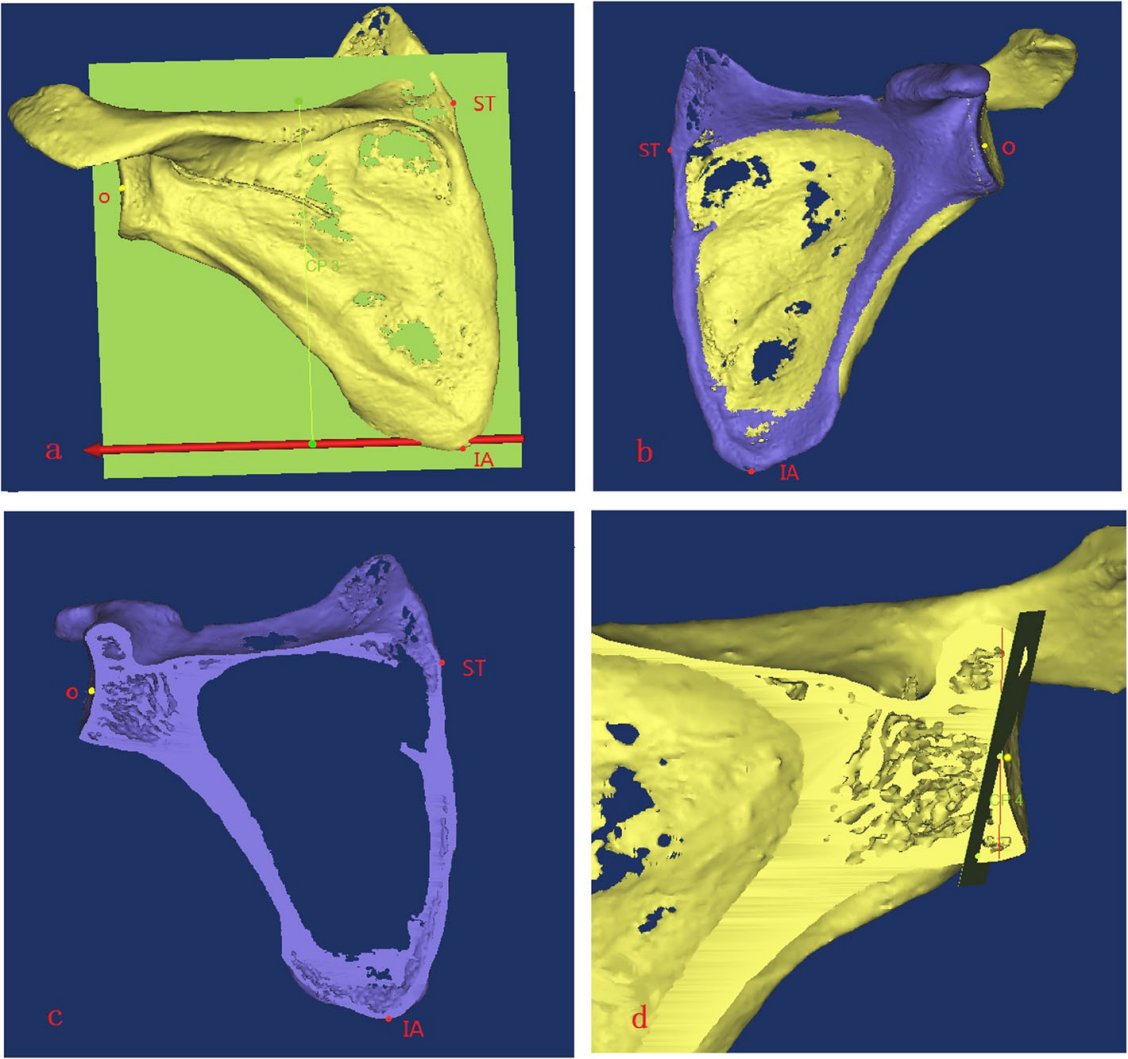
Determination of the coronal and osteotomy planes of the scapula

The coronal and osteotomy planes of the scapula were determined. The intersection point of the scapular spine and medial scapular edge was set as point ST, while the lower scapular angle was set as point IA. The plane formed by the connection of points O, ST, and IA was considered as the coronal scapular plane [14]. The scapula was split into two parts, with the coronal plane as the cutting plane (Fig. 3a–c). The vertical line and 10° inferior tilt line of the glenoid cavity on the coronal plane were determined, and a 10° inferior tilt section perpendicular to the coronal plane was created, which was the simulated osteotomy plane of the glenoid cavity (Fig. 3d).

#### Step d

**Fig. 4:**
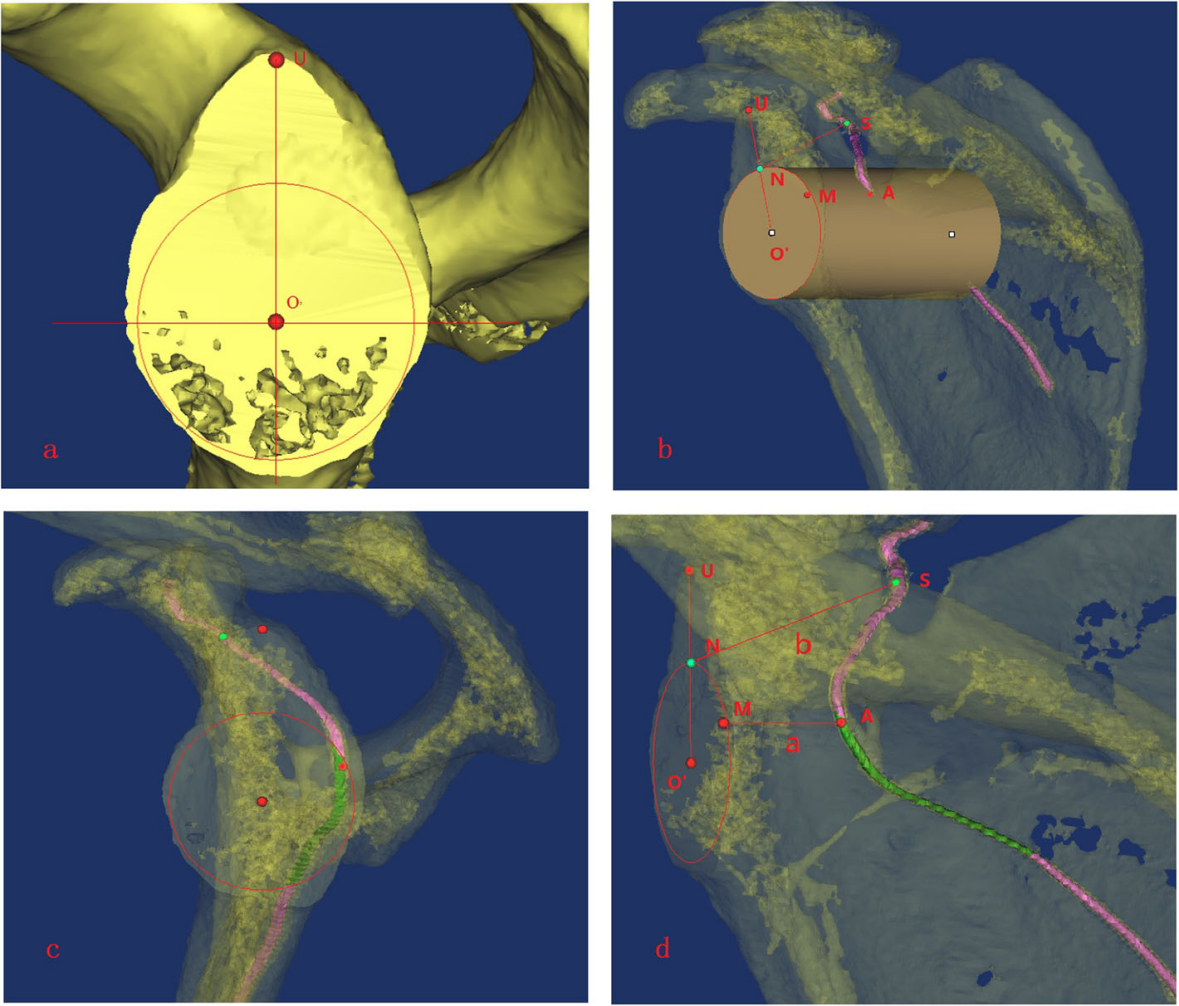
Determination of the dangerous areas and bone marking point

The baseplate position and size were determined. Taking the lower edge of the simulated osteotomy plane as the edge, the best fitting circle was designed. This circle was the baseplate, while the circle diameter was the baseplate diameter (*d*). The highest section point was point U, the circle center was point O, and the connecting line of points U and O was the longitudinal axis. A vertical line was created through point O, which was the horizontal axis, and the coordinate system of the base was determined (Fig. 4a) [15].

#### Step e

The dangerous and safe areas were determined. The scapula was made transparent (transparency level, 75%). The suprascapular nerve was projected vertically to the osteotomy plane. The position where the projection fell within the circle was the dangerous area (green), while the area not projected was the safe area (purple). A vertical screw placement in the dangerous area would cause suprascapular nerve injury (Fig. 4b, c).

#### Step f

The bone marking point was determined. The scapula was made transparent (transparency level, 75%). The closest point of the suprascapular nerve was point A, the closest point projected to the osteotomy plane was point M, the suprascapular nerve at the scapular notch was point S, and the intersection point of the longitudinal axis of the coordinate system and circle was point N (Fig. 4d).

#### Step g

**Fig. 5:**
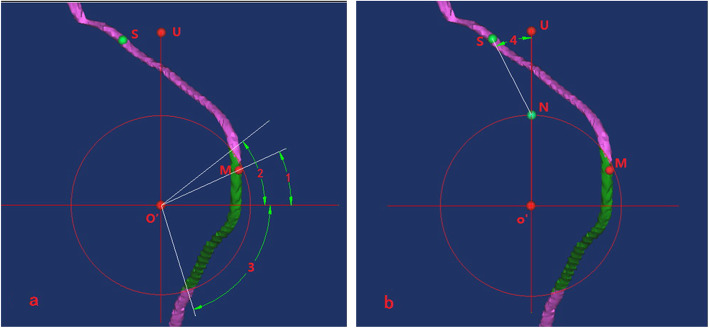
Measurement of the data related to the coordinate system

The data correlated to the coordinate system were measured. The distance from the nearest point of the nerve to the osteotomy plane (*a*), the distance from the scapular notch to the upper baseplate edge (*b*), the angle between the projection point of the nearest point of the nerve and coordinate horizontal axis (∠1), the angle between the projection point of the upper point of the dangerous area and coordinate horizontal axis (∠2), the angle between the projection point of the lower point of the dangerous area and coordinate horizontal axis (∠3), and the angle between the line connecting the suprascapular nerve notch to the upper baseplate edge (∠4) were measured (Fig. 5a, b).

In order to reduce errors, two persons performed the measurements and modeling of each specimen after scanning, and a third person recorded and summarized it. The average value was taken as the final data of each specimen.

### Statistical analysis

The software program SPSS 21.0 (SPSS, Chicago, IL, USA) was used to perform the statistical analysis. Continuous variables of normal distribution were expressed as mean ± standard deviation, continuous variables of non-normal distribution were expressed as median (interquartile range [IQR]), and categorical variables were expressed as frequency (percentage [%]). The correlation among continuous variables was analyzed using Pearson correlation coefficients. A *P* value of < 0.05 was considered statistically significant.

## Results

### General information

A total of 12 fresh adult cadaver shoulder specimens were dissected in the present study. Among these 12 fresh adult cadaveric specimens, five and seven specimens were left and right shoulder specimens, respectively.

### Measurement results

After the 3D reconstruction of the scapula and suprascapular nerve, the simulated osteotomy was performed, the bone marking points were marked, the corresponding distances and angles were measured, and the statistical analysis was performed. The data on the scapular glenoid cavity height (*h*), glenoid cavity width (*w*), baseplate diameter (*d*), distance between the nearest point of the suprascapular nerve and osteotomy plane (*a*), and distance between the suprascapular nerve notch and upper baseplate edge (*b*) are presented in Table 1.

**Table 1:** Measurements of bone marking points (*n* = 12)

	*h* (mm)	*w* (mm)	*d* (mm)	*a* (mm)	*b* (mm)
Mean	35.89	25.84	24.73	15.38	34.66
SD	2.20	1.64	1.56	2.02	2.41
Min	32.00	23.53	23.20	12.41	30.63
Max	39.86	29.10	27.80	18.71	38.93

*SD* standard deviation, *Min* minimum, *Max* maximum

### Pearson correlation analysis

The Pearson correlation coefficients among various distance parameters are presented in Table 2. The baseplate diameter was correlated with the glenoid cavity height and width, and the Pearson correlation coefficients were 0.603 (*P* = 0.038) and 0.899 (*P* = 0.000), respectively. The distance from the nearest point of the nerve to the osteotomy plane was highly correlated to the distance from the nerve at the scapular notch to the upper baseplate edge, with a Pearson correlation coefficient of 0.725 (*P* = 0.008). However, these two parameters were not correlated with the glenoid cavity height and width (both, *P* > 0.05).

**Table 2:** Pearson correlation coefficient among various distance parameters (*n* = 12)

	*h* (*P* value)	*w* (*P* value)	*d* (*P* value)	*a* (*P* value)	*b* (*P* value)
*h*	1	0.742** (0.006)	0.603* (0.038)	− 0.123 (0.704)	0.232 (0.468)
*w*	0.742** (0.006)	1	0.899** (0.000)	− 0.353 (0.260)	0.195 (0.543)
*d*	0.603* (0.038)	0.899** (0.000)	1	0.241 (0.451)	0.322 (0.307)
*a*	− 0.123 (0.704)	− 0.353 (0.260)	0.241 (0.451)	1	0.725** (0.008)
*b*	0.232 (0.468)	0.195 (0.543)	0.322 (0.307)	0.725** (0.008)	1

**Significantly correlated at 0.01 level (bilateral)

*Significantly correlated at 0.05 level (bilateral)

The data for ∠1, ∠2, ∠3, and ∠4 are presented in Table 3. The Pearson correlation coefficients among the bone marking point angles are presented in Table 4. There was a correlation between angles 1 and 2, and the Pearson correlation coefficient was 0.610 (*P* = 0.035). However, there was no correlation among the other angles (all, *P* > 0.05).

**Table 3:** Measurements of bone marking point angles (*n* = 12)

	∠1 (°)	∠2 (°)	∠3 (°)	∠4 (°)
Mean	27.33	15.17	53.75	21.67
SD	7.96	13.76	16.01	13.27
Min	12.00	− 3.00	26.00	6.00
Max	38.00	38.00	76.00	46.00

**Table 4:** Pearson correlation coefficient among bone marking point angles (*n* = 12)

	∠1 (*P* value)	∠2 (*P* value)	∠3 (*P* value)	∠4 (*P* value)
**∠**1	1	0.610* (0.035)	− 0.079 (0.808)	− 0.051 (0.876)
**∠**2	0.610* (0.035)	1	− 0.044 (0.892)	0.337 (0.284)
**∠**3	− 0.079 (0.808)	− 0.044 (0.892)	1	0.098 (0.761)
**∠**4	− 0.051 (0.876)	0.337 (0.284)	0.098 (0.761)	1

*Significantly correlated at 0.05 level (bilateral)

## Discussion

Reverse shoulder arthroplasty is important for treating various severe shoulder joint diseases. Glenoid cavity osteotomy and baseplate fixation are important procedures in reverse shoulder arthroplasty. During the baseplate fixation in surgery, the screw may lead to suprascapular nerve injury, which would cause shoulder joint pain and weakness [16].

Neither a simple cadaver study [17] nor a simple shoulder joint CT [18] can directly reveal the 3D relationship between the scapula and suprascapular nerve, which limits the research. Hence, cadaveric dissection, nerve marking, and CT were combined. After processing using the Mimics software, the 3D relationship between the scapula and suprascapular nerve could be clearly displayed, and the glenoid cavity osteotomy and baseplate implantation could be simulated. This allowed for the accurate measurement and preoperative planning, thereby avoiding the complications of nerve injury.

### Placement position and baseplate size in reverse shoulder arthroplasty

During the shoulder adduction after reverse shoulder arthroplasty, the impact of proximal humeral prostheses on the lower edge of the scapular neck produces scapular notching, which leads to joint instability and aseptic inflammation. In the studies conducted by Boileau et al. [19] and Sirveaux et al. [20], the incidence of scapular notching reached 53% and 67%, respectively, at 6 months after surgery. Biomechanical studies on the position of the prosthetic baseplate revealed that the placement of the glenoid cavity baseplate should be as close as possible to the lower glenoid cavity edge and that a 10–15° declination would further reduce the probability of impact [21]. The present study followed this principle when simulating the glenoid cavity osteotomy. The osteotomy plane, which inclined downward by 10°, was created on the sagittal plane perpendicular to the coronal plane, in order to simulate the prosthetic baseplate placement at the lower section edge.

There have been concerns regarding whether the models of presently available commercial prosthetic materials are too large for Asian populations. Furthermore, baseplates with excessively large diameters would cause the micromotion of glenoid cavity prostheses and less impingement-free range of motion, which would likely lead to early prosthetic loosening [22]. Therefore, it is especially important to investigate prosthesis models that are more suitable for Chinese patients. In the present study, the average glenoid cavity height and width were 35.89 mm and 25.84 mm, respectively, and the glenoid cavity height was highly correlated with the glenoid cavity width. Conversely, the glenoid cavity height and width of other populations were as follows: 39.5 and 31.0 mm in the USA [23], 41.3 and 29.4 mm in France [24], 31.5 and 23.1 mm in Japan [25], and 36.6 and 27.8 mm in Switzerland [26], respectively. Those in China were significantly different from those in other countries. In the present study, the baseplate diameter of Chinese patients was correlated to the glenoid cavity height and width but was more strongly correlated with the glenoid cavity width. Therefore, evaluating the baseplate diameter based on the glenoid cavity width would be more reliable.

### Safe area for screw placement and screw length

In the reverse shoulder arthroplasty, the bottom plate of the glenoid cavity baseplate is usually made of a metal plate, which is fixed on the pre-ground osteotomy surface of the glenoid cavity without bone cement fixation, and only uses screws [27]. Baseplates fixed using bicortical screws are significantly more stable than bases with screw tips in the cancellous bone. In clinics, surgeons usually use two or four screws to fix the glenoid cavity baseplate. Based on the shape of the scapula and glenoid cavity, the anterior coracoid process base edge and lateral scapular edge have dense bones (the bone density was determined by the CT value, and the upper and inferior screw tracks were all in the dense bone), which are the common superior and inferior screw fixation positions. The suprascapular nerve runs posteriorly and laterally through the scapular notch and turns to the posterior medial side after passing through the spinoglenoid notch. Therefore, superior and posterior screws may cause suprascapular nerve injury, while anterior and inferior screws would not cause nerve injury.

The safe suprascapular nerve area was previously measured by cadaver study, and this was evaluated by screwing screws into the body and observing the nerve injury. In reverse shoulder arthroplasty, posterior screws have greater risks in terms of baseplate fixation (e.g., suprascapular nerve trunk and glen humeral joint branch injuries), when compared to superior screws. Hart et al. [12] reported that the posterior screw damaged the suprascapular nerve and artery. Hence, it was suggested that the superior and inferior screws can be fixed with the greatest length, while the posterior screw has a danger of damaging the suprascapular nerve. The evaluated dangerous area was the area behind the connection line between the supraglenoid and infraglenoid tubercles. Yang et al. [18] re-evaluated the safe area according to the spinoglenoid notch position by scanning the scapula and modified the dangerous area by referring to the right shoulder and locating it between the 2 and 8 o’clock positions, according to the clock indication system.

In the present study, the suprascapular nerve marked using the barium thread was located at the original anatomical position, and the 3D images were displayed by CT and reconstruction, which were more objective. After the simulated osteotomy, the dangerous area for posterior screw placement was evaluated as the area where the angle between the upper edge and transverse axis exceeds 38°, the angle between the lower edge and transverse axis exceeds 76°, and the angle between the projection point of the nearest point of the nerve at the scapular notch and transverse axis is 12.00–38.00°. The length of the posterior screw placed in this area should be shorter than the distance between the nearest point of the nerve and the osteotomy plane. It is relatively safe to place the superior screw within these angles and lengths.

Since the anterior and inferior screws are located in the safe area for nerves, these would not cause damage to the suprascapular nerve. However, this does not indicate that the screw length is not limited in the safe area. Indeed, long screws would cause wear to the subscapular muscle, which would lead to fat infiltration and fibrosis [28, 29]. Hence, the screw length should be determined based on the depth of the borehole in the bone.

In the present statistical analysis, the Pearson correlation coefficient between angles 1 and 2 was 0.610, which indicates that the closest point of the suprascapular nerve was close to the upper edge of the dangerous area. Therefore, when placing the posterior screw, the screw tip should not face the upper edge as far as possible, even when the screw is not placed vertically. Otherwise, this would make the screw tip close to the closest point of the nerve and increase the nerve injury risk.

The distance between the nearest point of the nerve and osteotomy plane (*a*) and between the nerve at the scapular notch and upper baseplate edge (*b*) had no correlation with the glenoid cavity height (*h*) and width (*w*), since the *P* values of the Pearson correlation coefficients were both greater than 0.05. Furthermore, the superior and posterior screw lengths were not correlated with the glenoid cavity height and width. The correlation coefficient between the distance between the nearest nerve point and osteotomy plane (*a*) and between the nerve at the scapular notch and upper baseplate edge (*b*) was 0.725. This indicates that the lengths of these two screws have a close positive correlation and that the length of the posterior screw is proportional to the length of the superior screw.

### Retractor placement during surgery

During the grinding and osteotomy of the glenoid cavity in reverse shoulder arthroplasty, a shoehorn retractor must be placed to completely expose the glenoid cavity, clear the surgical field, and reduce the damage of the grinding drill to the surrounding tissues. The position and tension of the retractor can cause compression on the suprascapular nerve. Based on the 3D anatomical relationship between the scapula and suprascapular nerve in the present study, the nearest point of the nerve at the spinoglenoid notch (point A, including the 12.00–38.00° with the transverse axis) was the most dangerous area. The medial side of the suprascapular nerve at this point was the scapular spine. However, there was no soft tissue buffer after the nerve was compressed, which can easily cause suprascapular nerve injury. Distant from the spinoglenoid notch (point A), the supraspinatus or infraspinatus can be found at the medial side of the suprascapular nerve. The supraspinatus or infraspinatus has a soft tissue buffer. Thus, the risk of injury when being pulled was smaller. Therefore, when placing the retractor hook, placing this at the spinoglenoid notch (including the 12.00–38.00° with the transverse axis) should be avoided.

In response to the risk of suprascapular nerve injury in reverse shoulder arthroplasty, shoulder joint anatomical designing, suprascapular nerve marking, CT, and Mimics 3D reconstruction were performed to present the 3D anatomical relationship between the suprascapular nerve and scapula by simulating the osteotomy and baseplate placement. Furthermore, the correlation between the baseplate size and glenoid cavity height and width was clarified. In clinics, baseplates that are more suitable for the glenoid cavity size of Chinese patients must be selected. In addition, the safe area for placing screws for the glenoid cavity base, the safe distances for such screws, and the dangerous area for placing retractors were also clarified, providing an important reference for suprascapular nerve protection during reverse shoulder arthroplasty.

It could be summarized from this study that the baseplate diameter strongly correlated with the glenoid cavity width, the dangerous area for posterior screw placement was at the angle between the upper edge and transverse axis exceeding 38° and between the lower edge and transverse axis exceeding 76°, the suitable angle between the superior screw and longitudinal axis was 21.67 ± 13.27°, the suitable superior screw length was 34.66 ± 2.41 mm, and the angle between the projection point of the nearest point and transverse axis was 27.33 ± 7.96°, which was the dangerous area for retractor placement. The distance between the nearest point of the nerve and osteotomy plane was 15.38 ± 2.02 mm. These data provide an important reference for the selection of base size during RTSA and protection of suprascapular nerve during base fixation.

### Limitations

There were still several limitations to the present study. First, the sample size was limited. Since donated samples are precious and not easily available, the limited sample size may result in unrepresentative data. Second, in the present study, the muscle tension of the cadaveric specimen was changed, which may have changed the anatomical position of the nerve, and inevitably lead to inaccurate measurements.

## Conclusion

When performing reverse total shoulder arthroplasty, the baseplate size should be correlated with the glenoid cavity width. The relationship between the screw and suprascapular nerve and retractor placement position should be carefully considered, in order to avoid damaging the suprascapular nerve.

## Data Availability

The datasets used and/or analyzed during the current study available from the corresponding author on reasonable request.

## Abbreviations

3D: Three-dimensional
RTSA: Reverse total shoulder arthroplasty
CT: Computed tomography
